# The CHALO! 2.0 mHealth-Based Multilevel Intervention to Promote HIV Testing and Linkage-to-Care Among Men Who Have Sex with Men in Mumbai, India: Protocol for a Randomized Controlled Trial

**DOI:** 10.2196/59873

**Published:** 2024-11-05

**Authors:** Jatin Chaudary, Shruta Rawat, Alpana Dange, Sarit A Golub, Ryung S Kim, Venkatesan Chakrapani, Kenneth H Mayer, Julia Arnsten, Viraj V Patel

**Affiliations:** 1 The Humsafar Trust Research Unit Mumbai India; 2 Department of Psychology Hunter College City University of New York New York, NY United States; 3 Department of Epidemiology and Population Health Albert Einstein College of Medicine Bronx, NY United States; 4 Centre for Sexuality and Health Research and Policy Chennai India; 5 The Fenway Institute Fenway Health Boston, MA United States; 6 Department of Medicine Beth Israel Deaconess Medical Center Harvard Medical School Boston, MA United States; 7 Division of General Internal Medicine, Department of Medicine Albert Einstein College of Medicine Montefiore Health System Bronx, NY United States

**Keywords:** Keywords: digital health, HIV prevention, social media, stigma, gay, men who have sex with men, MSM

## Abstract

**Background:**

Current programs to engage marginalized populations such as gay and bisexual individuals and other men who have sex with men (MSM) in HIV prevention interventions do not often reach all MSM who may benefit from them. To reduce the global burden of HIV, far-reaching strategies are needed to engage MSM in HIV prevention and treatment. Globally, including low- and middle-income countries, MSM are now widely using internet-based social and mobile technologies (SMTs; eg, dating apps, social media, and WhatsApp [Meta]), which provides an unprecedented opportunity to engage unreached and underserved groups, such as MSM for HIV prevention and care.

**Objective:**

This study aimed to assess the effectiveness of a multilevel mobile health (mHealth)–based intervention to improve HIV testing uptake and status neutral linkage-to-care among sexually active MSM reached through internet-based platforms in Mumbai, India.

**Methods:**

In this randomized controlled trial, we will determine whether CHALO! 2.0 (a theory-based multilevel intervention delivered in part through WhatsApp) results in increased HIV testing and linkage-to-care (prevention or treatment). This study is being conducted among 1000 sexually active MSM who are unaware of their HIV status (never tested or tested >6 months ago) and are recruited through SMTs in Mumbai, India. We will conduct a 12-week, 3-arm randomized trial comparing CHALO! 2.0 to 2 control conditions—an attention-matched SMT-based control (also including a digital coupon for free HIV testing) and a digital coupon–only control. The primary outcomes will be HIV testing and status neutral linkage-to-care by 6 months post enrollment. Participants will be followed up for a total of 18 months to evaluate the long-term impact.

**Results:**

The study was funded in 2020, with recruitment having started in April 2022 due to delays from the COVID-19 pandemic. Baseline survey data collection began in April 2022, with follow-up surveys starting in July 2022. As of April 2022, we enrolled 1004 participants in the study. The completion of follow-up data collection is expected in January 2025, with results to be published thereafter.

**Conclusions:**

While global health agencies have called for internet-based interventions to engage populations vulnerable to HIV who are not being reached, few proven effective and scalable models exist and none is in India, which has one of the world’s largest HIV epidemics. This study will address this gap by testing a multicomponent mHealth intervention to reach and engage MSM at high priority for HIV interventions and link them to HIV testing and prevention or treatment.

**Trial Registration:**

ClinicalTrials.gov NCT04814654; https://clinicaltrials.gov/study/NCT04814654. Clinical Trial Registry of India CTRI/2021/03/032280

**International Registered Report Identifier (IRRID):**

DERR1-10.2196/59873

## Introduction

### Background

HIV remains a significant public health issue, with an estimated 39 million individuals living with HIV in 2021 and new diagnoses continuing to be concentrated among key populations globally including gay, bisexual, and other men who have sex with men (MSM) [1]. India has the third largest population of people living with HIV globally, with it disproportionately affecting MSM. In India, MSM have an estimated national HIV prevalence of 3.78% compared with 0.21% among the overall population in 2021 [2] and are designated a key priority population for HIV interventions by India’s National AIDS Control Program. To end the HIV epidemic, one of the initial steps includes HIV testing and enabling linkage to treatment (which is free for all in India) or prevention. However, HIV testing remains suboptimal among Indian MSM as it does globally. Previous studies, that recruited Indian MSM through in-person approaches, report that 18%-49% of MSM have never been HIV-tested [3,4]. In recent studies reaching Indian MSM online, a substantial proportion (23%-47%) had never undergone HIV testing [5,6], and in 1 large study, approximately 75% of sexually active MSM were unaware of their current HIV status (ie, were never HIV-tested or were last tested more than 12 months before) [7].

HIV testing interventions and strategies for MSM are often limited in their reach and impact, in part due to the social stigma associated with both same-sex behaviors and HIV. Both globally and in India, MSM often face barriers in accessing accurate information about HIV and services tailored to their needs [8-11]. Coupled with community-level stigma, a lack of knowledge and support for accessing sexual health care reduces awareness of needing and motivation for HIV testing and subsequent care. However, a key challenge faced by outreach programs is how to engage individuals who fear being seen at MSM-identified spaces due to the stigma associated with attending an LGBTQ (lesbian, gay, bisexual, transgender, and queer or questioning) or HIV center (eg, general voluntary testing or counseling centers) or other logistical challenges. Traditional interventions that rely on an in-person outreach model may not address barriers that prevent individuals from seeking care and may also fail to reach MSM who do not frequent physical meeting venues, do not identify as a sexual minority, or are unlikely to go to LBGTQ- or HIV-affiliated settings [5-7,12-14]. Innovative strategies are thus needed to overcome stigma and ensure equitable and accessible services to meet the UNAIDS (Joint United Nations Programme on HIV/AIDS) 95-95-95 goals, that is, 95% of people living with HIV know their HIV status, 95% of those diagnosed receiving sustained antiretroviral therapy, and 95% of those receiving therapy achieving viral suppression by 2030 [8].

Evidence suggests that mass media HIV awareness campaigns, information provision, linkage to support services, stigma reduction, and reducing structural barriers may all improve HIV testing rates and subsequent linkage-to-care [15,16] mobile health (mHealth)–based interventions leveraging social media and other internet-based communication technologies (SMTs) such as WhatsApp [Meta] provides opportunities to both reach and engage MSM into health services at scale, although little evidence from India currently exists. SMT use has rapidly increased in both India and globally. There has been a rapid growth in India’s digital population with 600 million active internet users in the past decade, and approximately 487.5 million active WhatsApp users, making WhatsApp a critical communication tool in India [17]. This growth has been followed by an increasing use of SMTs by MSM and other groups to socialize and seek sexual, romantic, or transactional partners [18]. Thus, leveraging SMTs presents an unprecedented opportunity to reach and engage Indian MSM in HIV prevention, linkage to health care, and provide access to support services [13].

Current evidence suggest that internet-based interventions can influence offline health behaviors [8,13,15,19-21] including HIV testing. However, there are limited data assessing the potential of SMTs to increase HIV testing among MSM in India or South Asia aside from small pilot studies [7,16,18,19,21-23]. Previous research among Indian MSM suggest that interventions using SMTs are feasible and acceptable [5,14]. However, there are limited data on its effectiveness for increasing HIV testing in India or other South Asian countries. One pilot study among Indian MSM used SMTs to deliver HIV-testing and prevention-related messages to MSM recruited through social media, finding high feasibility, acceptability, and a significant rise in self-reported HIV testing (from 31.5% to 43.8%) and an increased intention to test (from 44.6% to 65.4%) over 12 weeks [7]. However, it did not objectively assess HIV testing, address structural barriers to testing access, or assess linkage to care (important elements to inform widespread scale-up). Because changing health behaviors, especially for stigmatized conditions could be a slow process, evaluating outcomes over a longer duration of time is warranted to inform HIV-related outreach strategies, although some data exist globally and to our knowledge, none in India.

### Aims

First, to determine whether CHALO! 2.0 increases HIV testing compared with control conditions and to identify factors associated with HIV testing.

Second, to determine whether CHALO! 2.0 increases linkage-to-care (both HIV prevention through counseling and use of pre-exposure prophylaxis [PrEP] and HIV treatment with antiretrovirals) and to identify factors associated with linkage to care.

### Objectives

The objective of this study is to test CHALO! 2.0 (a WhatsApp-based multilevel intervention) in a randomized control trial to help answer the following questions: (1) Is CHALO 2.0 efficacious for increasing actual (not self-reported) HIV testing, compared with an attention-matched control (AMC)? (2) Does CHALO! 2.0 results in durable increases in repeat HIV testing? (3) Does CHALO! 2.0 improve linkage to both prevention and treatment? (4) If offered multiple free testing choices (at MSM community organizations, through private laboratories, or self-testing) without a behavioral intervention, do MSM obtain HIV tests and link to care, or is an active SMT behavioral intervention needed to increase uptake?

Our hypothesis is that CHALO! 2.0, a community-developed, theoretically grounded, SMT-based intervention, will reach and engage Indian MSM, enhance motivation and skills to obtain HIV testing and care, and overcome structural barriers compared with either attention-matched or digital coupon–only (DCO) control arms. The rationale is that determining the efficacy of a rapidly scalable SMT-based intervention is needed to facilitate widespread adoption. Even a modestly effective SMT-based intervention reaching thousands of MSM can dramatically and significantly impact the epidemic by increasing HIV status awareness and linkage-to-care and thus reducing new infections.

## Methods

### Trial Design

This study is a 3-armed, randomized controlled trial among 1000 MSM, recruited through SMTs and unaware of their HIV status. Eligible participants are randomized 1:1:1 to 3 arms: (1) CHALO 2.0: an HIV-focused multimedia messaging arm to deliver information, motivation, and skills to access testing and care; (2) an AMC focused on general health messaging; and (3) a no message control arm. Participants in all arms receive digital coupons for choices of free HIV testing and access to virtual peer outreach workers for linkage to care support. Active messaging occurs twice per week for 12 weeks with follow-up assessments and outcome measurement through 18 months post enrollment. This design allows all participants to receive intervention components, consistent with ethical and accepted practice.

### Study Setting

The study is being implemented in Mumbai and Thane, Maharashtra, India, by The Humsafar Trust (HST) in collaboration with academic partners. Established as one of India’s first LGBTQ+ (lesbian, gay, bisexual, transgender, queer or questioning, plus [others]) community–based organizations, HST implements a variety of HIV-related prevention, support, and treatment programs across India and has a long history of academic research partnerships including its own Federal Wide Assurance registered institutional review board (IRB) [7,24].

Mumbai is a mega-metropolitan city in Maharashtra state with a population of 21.67 million in 2024 [25]. Thane is the most populous district in the state of Maharashtra, situated contiguously with Mumbai, with individuals commuting to and from both locations for work, school, residence, and socialization. Recent reports from India’s National AIDS Control Organization (NACO) show that Maharashtra is estimated to have the highest new HIV infections and an HIV prevalence of 1.2% in Mumbai and 0.46% in Thane (compared with 0.21% nationally in 2021), with new diagnosis concentrated among key populations including MSM [2].

### Inclusion and Exclusion Criteria

Inclusion criteria for this study are (1) age of ≥18 years; (2) live or work in the Mumbai metropolitan area, including Thane; (3) are fluent in Hindi or English, (4) male sex at birth; (5) report having anal sex with men in the past 1 year; (6) report either that they have never tested for HIV, are unaware of their HIV test results, or are HIV-negative with their last HIV test >6 months ago and have had anal sex since last test; (7) able to provide and verify their WhatsApp mobile number; and (8) have not participated in a previous HIV research study (by self-report). The exclusion criteria include being unwilling to consent to the study, failing to verify the provided WhatsApp number, or not completing the baseline survey. Sexual orientation or expression was not part of the eligibility criteria. This study did not include transgender men, as it focused on cisgender MSM and not transgender men. As a quality control measure, individuals who provided duplicate digital payment ID (Google Pay, Paytm, or UPI [Unified Payments Interface] ID) or submitted the survey in less than 3 minutes will be excluded from the analyses.

### Theoretical Frameworks for CHALO! 2.0

**Figure 1:**
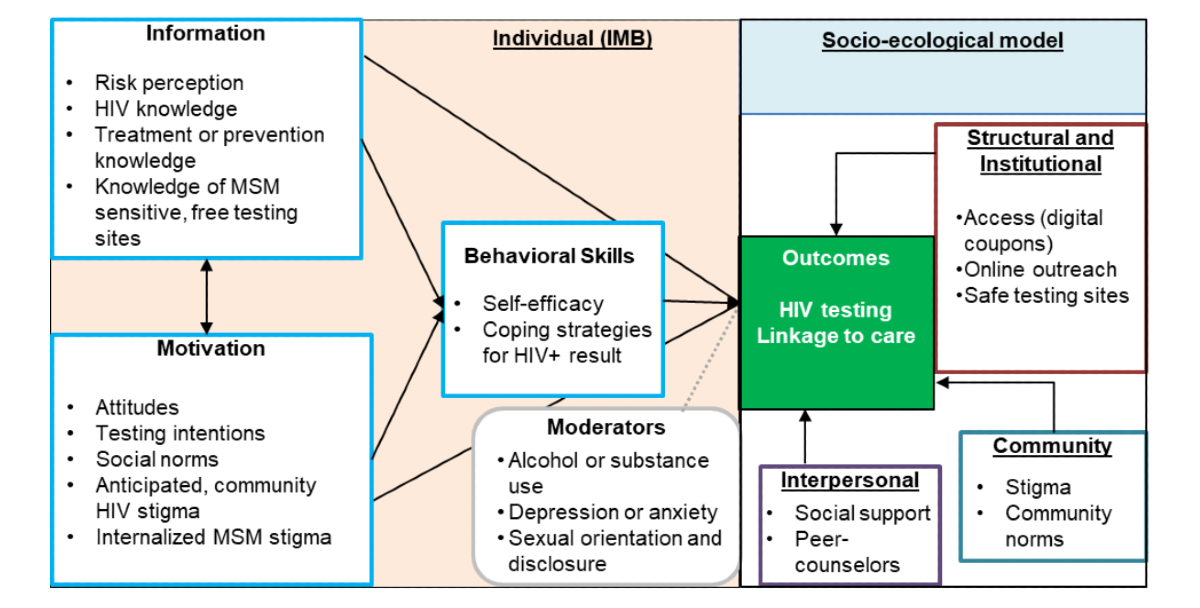
CHALO! 2.0 conceptual model based on information-motivation-behavioral and socio-ecological model (SEM) frameworks. IMB: information-motivation-behavioral. MSM: men who have sex with men.

CHALO! 2.0 is developed as a multicomponent multilevel intervention, grounded in the socio-ecological model (SEM) [26,27] and the information-motivation-behavioral skills (IMB) frameworks for behavior change [28-31] (Figure 1). The SEM demonstrates the need to target factors at various levels of influence to support behavior change, while the IMB model focuses on information, motivation, and behavioral skills as determinants for HIV testing and linkage-to-care. CHALO! 2.0 intervention components were designed to target these domains as shown in Figure 1.

### The CHALO! 2.0 Intervention

**Figure 2:**
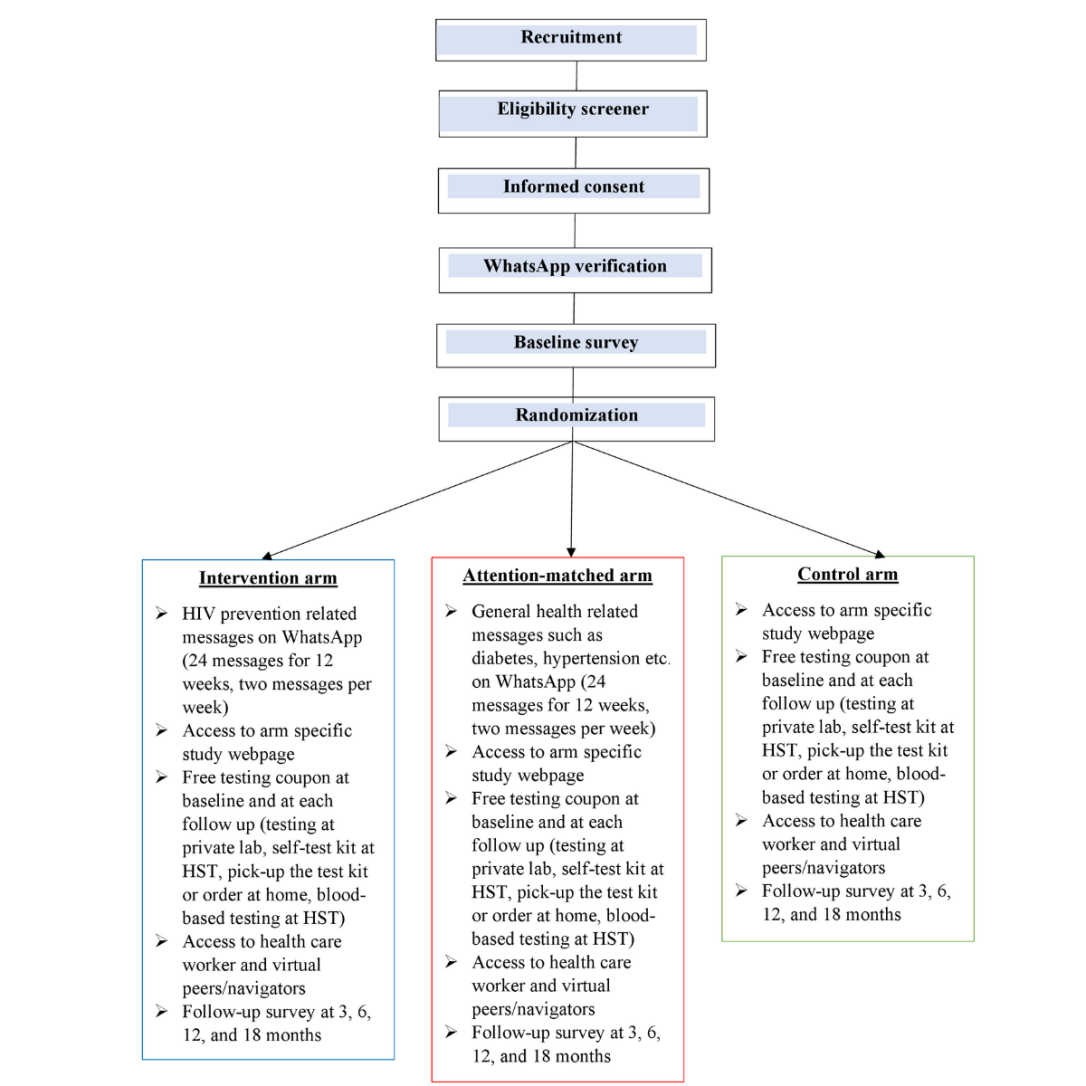
Participant flow and components under each study arm. HST: The Humsafar Trust.

Participants randomized to this arm receive a 12-week intervention comprising digital media messages sent twice weekly (24 unique messages) through HST’s verified WhatsApp business account (Figure 2). These messages are focused on topics related to HIV, HIV testing, and HIV prevention (including PrEP) and treatment specifically tailored for Indian MSM. The messages target information, motivation, and behavioral skills as well as community norms, stigma related to HIV, and same-sex attraction using a combination of text messages, infographics, and brief videos (eg, 1 minute in length).

The content of the messages served to provide the following:

1. Information: dissemination of HIV knowledge, logistical details about testing and care, and information about PrEP and postexposure prophylaxis (PEP).

2. Motivation: inclusion of brief video clips aimed at creating social norms for HIV testing.

3. Behavioral skills: presentation of video clips demonstrating how to request and obtain an HIV test at HST or a private laboratory using digital coupons, and guidance on ordering and using the HIV self-test kit.

4. Interaction: access to peer outreach and health care workers through WhatsApp messages and audio calls for resolving any queries and testing or linkage support.

### Intervention Content Development

We used a collaborative process with a multidisciplinary team to develop and refine all intervention components and study procedures over 5 months, similar to the CHALO pilot [13]. The team consisted of 14 individuals—3 community-based HIV researchers, a community advisory board (CAB), peer outreach workers, and HIV testing staff members. The intervention content underwent development and refinement through a series of 4 consultations with the community and peer outreach staff at HST. The workshops were led by 2 study team members (JC and SR) with expertise in group facilitation to identify the most important barriers and facilitators to HIV testing and preventive services, develop messages, and establish the sequence and scheduling of the messages or themes. A total of 14 MSM with diverse lived experiences participated in the workshops (eg, community-based organization clients, peer outreach workers, and HIV testing counselors). We used user-centered design and open-space technology processes to conduct the workshops, similar to the pilot study [13]. The open-space technique is a process that is used across disciplines to help ensure the inclusion of diverse viewpoints and has been used to facilitate the identification of challenges or barriers to a task or behavior (eg, HIV testing) and the identification of potential solutions to overcome them [32]. The workshop participants also identified top health priorities within the MSM community, apart from HIV, to be included in the AMC arm. The outcomes from the community consultations were then discussed with the CAB and the overall study team to finalize intervention messaging, sequence, and frequency of the messages. We leveraged videoconferencing tools to facilitate workshops (eg, breakout rooms and polling tools to reach consensus), considering COVID-19 restrictions on in-person meetings.

### Attention-Matched Control Arm

Participants randomized to the AMC arm receive informational messages disseminated twice a week for 12 weeks through the same WhatsApp Business account used for the intervention. These messages include both text and infographics. While the message delivery schedule aligns with the CHALO! 2.0 arm, the content in the AMC arm differs significantly. It focuses on addressing the top health priorities identified by participants in the community consultation, such as diabetes, hypertension, obesity, nutrition, stress management, and hair and skin care, among others. The messaging content for the AMC arm was finalized with guidance from the study team and the CAB.

### Digital Coupon–Only Control

Participants randomized to this arm receive only a welcome message with no health-related content.

### Common Components Across All Trial Arms

#### Personalized Digital Coupon for Free HIV Testing

All participants, regardless of their assigned arm, receive a personalized digital coupon after enrollment. The coupon comprises a code linked to their study ID. The digital coupon code is associated with participants’ mobile numbers at the back end, enabling the study team to reach out if preventive or treatment services are required. Each coupon is redeemable once during the study period. The participants will receive the study coupon at baseline and at each follow-up.

#### Choice of HIV Testing Strategy

Participants are offered digital coupons for free testing and can select from three testing modalities, such as (1) standard counselor-based finger-prick rapid testing at HST (3 sites across Mumbai); (2) self-testing using rapid oral swabs (Orasure, Inc), which can be used at any of the HST’s 3 sites, picked-up by participants or delivered through a local courier to participant’s preferred location; and (3) a fourth generation HIV antibody/antigen test at a private laboratory with locations across Mumbai and Thane. Participants walking in to test (either blood-based or oral swab kit) are provided with face-to-face pre-and posttest counseling by the counselor and health care workers (HCWs). Participants opting for tests using the self-test kit or at a private laboratory can watch the pre- and posttest counseling and “how to use the kit” video on the study web pages.

#### Informational Web Page

Each participant receives an arm-specific link to an identical informational web page, detailing testing sites, the contact information of online peer counselors, and MSM-specific health services provided by HST. This page hosts pre- and posttest counseling videos in case testing using the self-test kit at a preferred location or at a private laboratory and offers contact details for online peer counselors and HCWs.

#### Virtual Peer Outreach Workers

All participants have access to virtual peer outreach workers through WhatsApp to address inquiries related to testing facilities or self-test kit ordering or use, and linkage to services. The virtual peers also connect the participants with the HCWs.

#### Linkage-to-Care

Specific protocols are implemented for HIV-positive results, including linkage and monitoring. Participants are offered linkage to care services, regardless of their test result, and those testing positive are further provided with automated messages on positive living.

### Recruitment Strategies and Enrollment

The recruitment process involves various strategies such as outreach on MSM dating apps, advertisements by community-based Instagram (Meta) influencers, paid advertisements on Grindr (Grindr Inc), and the snowball technique. All advertisements contain a study link that directs potential participants to a landing page with more information about the study and an eligibility screener. Eligible participants proceed to an informed consent page on the Health Machine (Avegen), a web-based survey platform created for the study. After consenting, participants verify their WhatsApp number through a link sent from the study’s WhatsApp account and then proceed to the baseline survey followed by randomization as shown in Figure 2.

### Randomization and Blinding

Participants are randomized in a 1:1:1 ratio to the 3 arms—CHALO! 2.0 (intervention), AMC, and digital coupon (coupon-only)—using a random blocked design with a fixed block size of 6. Stratification is based on previous HIV testing history, that is, never tested or not tested in over 6 months. Allocation to the arms is automated through a system developed by the technology partner, reducing bias in allocation and analysis. To minimize bias, double blinding is used. Participants remain unaware of their assigned arm and receive automated messages for surveys. Staff collecting HIV testing and linkage-to-care data remain blinded. HST’s online peer counselors and navigators are unaware of the study hypothesis to maintain blinding.

### Online Study Assessments

Online surveys in Hindi and English collect self-reported data on sexual behaviors, previous HIV testing, HIV knowledge (transmission, treatment, and prevention), self-efficacy for HIV testing and care, stigma, and covariates as detailed in Table 1. Surveys also help assess potential contamination between the study arms. Participants receive automated baseline and then follow-up surveys at 3, 6, 12, and 18 months after enrollment. Participants receive WhatsApp reminders 3 and 7 days after the survey link is triggered. Nonresponses within 7 days of the baseline survey lead to baseline archiving. Follow-up surveys allow 1 month for completion.

**Table 1:** Domains assessed at baseline and follow-up surveys.

Survey section			Baseline		3 months		6 months		12 months		18 months
**Section 1: HIV testing access or preferences and PrEP^a^ or PEP^b^ use** [5,7,33-35]											
	HIV testing, HIV testing access	✓		✓		✓		✓		✓	
	Preferences for HIV testing, self-testing acceptability	✓		✓		✓		✓		✓	
	Intention to test in next 3 months	✓		✓		✓		✓		—^c^	
	Decisional balance for HIV testing—pros and cons	✓		—		—		—		—	
	HIV prevention knowledge, HIV testing knowledge	✓		✓		✓		✓		✓	
	HIV testing and coping self-efficacy	✓		✓		—		✓		—	
	PrEP decision balance (benefit and stigma)	✓		✓		✓		✓		—	
	HIV risk perception	✓		✓		✓		✓		✓	
	History of health care provider–diagnosed STIs^d^	✓		✓		✓		—		—	
**Section 2: Behaviors** [5,7,36]											
	Relationships, sexual and behaviors	✓		✓		✓		✓		✓	
	Condom use	✓		✓		✓		✓		✓	
	Alcohol use	✓		✓		✓		—		—	
	Substance use	✓		✓		✓		✓		✓	
**Section 3: Social support, resilience, and resources** [37,38]											
	HIV and sexual health social support	✓		✓		✓		✓		—	
	Gay or LGBTQ^e^ community attachment	✓		—		—		✓		—	
	Connectedness to LGBTQ social media	✓		✓		✓		✓		✓	
**Section 4: Stigma and mental health** [39,40]											
	Anticipated HIV stigma for testing	✓		—		—		—		—	
	Internalized homonegativity	✓		✓		✓		—		✓	
	Depression, anxiety	✓		✓		✓		✓		—	
**Section 5: Identity and outness** [7]											
	Sexual identity, outness	✓		✓		✓		✓		✓	
	Education, income, living status	✓		—		✓		✓		✓	
	Previous use of HST^f^, allied sites	✓		—		—		—		—	
	Online or virtual HIV and sexual health support	✓		—		—		—		—	

^a^PrEP: pre-exposure prophylaxis.

^b^PEP: postexposure prophylaxis.

^c^Not applicable.

^d^STI: sexually transmitted infection.

^e^LGBTQ: lesbian, gay, bisexual, transgender, and queer or questioning.

^f^HST: The Humsafar Trust.

### Tracking and Retention

Participants are sent automated reminders on WhatsApp alongside their individual survey link for scheduled surveys at 3, 6, 12, and 18 months. If surveys are not completed, peer outreach workers attempt to contact participants up to 3 times by WhatsApp messages and phone calls.

### Outcomes

#### For HIV Testing

Primary: Verified receipt of an HIV test within 6 months post randomization.

Secondary: Frequency of HIV testing within 18 months, receipt of verified HIV tests within 12 and 18 months, and self-reported HIV tests within 6 months.

#### For Linkage to Care

Primary: Linkage-to-prevention (yes or no) comprising counseling or PrEP use.

Secondary: HIV treatment linkage, antiretroviral therapy initiation for HIV-positive persons, sexual behavior indicators, all self-reported through baseline and follow-up assessments.

#### Exploratory Outcomes

The exploratory outcomes include the use of alcohol or other substances, the use of PEP, testing or treatment for any sexually transmitted infections, and the type of care site access for testing and treatment (HST community-based organization vs other health care facilities).

Study arm-specific outcomes such as HIV knowledge, stigma measures, risk perception, self-efficacy, social support, intention to get an HIV test, and SMT engagement.

### Sources of Data

#### Survey Data

Self-reported data at Baseline and at subsequent follow-up surveys.

#### Testing Data

Use of the unique coupons at different testing sites, verified by peer counselors or laboratory technicians, recording dates, times, and locations of usage. The test results, whether from a self-test at home or pickup or testing at the private laboratory are shared by the participants, which are verified against the test report uploaded by the laboratory onto their secure portal with access to the data manager. Counselor-based testing is verified at HST by cross-referencing phone numbers with the clinic database. Self-test participants receive incentives for sharing pictures of the test kit dial.

#### Linkage to Care

HCWs oversee linkage to treatment clinics and primary care or PrEP access, providing information to the data manager.

#### Messaging Data

WhatsApp Business Analytics data shared by WhatsApp provide details on whether the messages are being sent, delivered, and read by the study participants. The online peer counselor interactions are monitored at the back end through a messaging platform. The web page visits and engagement with content are assessed by studying website visits using Google Analytics and analytics from the YouTube (Google) channel. Considering the limitation of attributing data to unique individuals due to technological constraints.

### Exit Interviews

To provide insights into why CHALO! 2.0 may or may not work to increase HIV testing and linkage-to-care and obtain data to inform potential scale-up, we will perform phone-based exit interviews with 5-10 randomly selected participants from each of the following groups, who are never HIV tested, tested only once, tested multiple times, linked to HIV treatment, linked to HIV prevention care (eg, PrEP and counseling), and not linked to care. After obtaining verbal consent, we will record the interviews, transcribe, and explore the interview data using thematic analysis.

### Statistical Analysis Plan

All primary analyses will be intention-to-treat, including participants who are lost to follow-up. In secondary analyses, we will conduct per-protocol analyses to ascertain outcomes among participants who viewed messages, identify exposure levels (“intervention dose”) that may be associated with outcomes, and compare models stratified by testing history. We will conduct additional exploratory analyses on a per-protocol basis to assess factors such as SMT engagement, usability, and interaction with intervention “dose.”

Aim 1 analyses will compare the proportion of HIV testing at 6 months between CHALO! 2.0 (n_1_=300) and control participants (n_2_=300) using chi-square tests. We will also compare CHALO! 2.0 participants with DCO participants using chi-square tests. Although this is a randomized trial, to account for possible confounders we will also perform logistic regression [41] with HIV testing at 6 months as the dichotomous outcome and intervention arm as the primary predictor, adjusting for the covariates. In secondary analyses, we will repeat these analyses with HIV testing at 12 months and 18 months as outcomes. We will also compare the cumulative frequency of HIV tests at 18 months between CHALO! 2.0 participants (n_1_=266) versus AMC (n_2_=266) or DCO (n_2_=266) using Poisson tests. We will account for possible confounders using Poisson regression [41], with frequency of HIV testing as the outcome and intervention arm as the primary predictor, adjusting for confounders.

Aim 2 analyses will compare rates of linkage-to-prevention (ie, counseling and PrEP initiation) at 12 months for HIV-negative participants between study arms using chi-square tests and will also perform logistic regression with linkage-to-prevention as the outcome, study arm as the primary predictor, and adjusting for covariates. For secondary and exploratory outcomes, we will use ANOVA for continuous variables and chi-square tests for categorical outcomes. Exploratory analyses will report initial HIV-testing strategies and assess associations with participant characteristics. Secondary and exploratory outcomes will be analyzed using appropriate statistical tests (ANOVA or chi-square) as needed.

### Sample Size

For aim 1, with a total of 1000 participants, we expect at least 900 participants at 6 months, and 800 participants at 12 months. This will provide a total of n=300 per arm at 6 months, and 266 participants per arm at 12 months. Based on findings from online intervention studies of HIV testing showing 1.7-2.2 increases in odds of testing in intervention versus control conditions (across low- and high-income countries and heterogeneous online interventions) [42-45], we estimate the proportion of HIV testing at 6 months to be 70% (210/300) for CHALO! 2.0 versus 35% (105/300) for AMC and 30% (90/300) for DCO. With the expected sample, the study’s 2 main effects (CHALO! 2.0 vs AMC and CHALO! 2.0 vs DCO) have >99% power (Table 2). We also provide power estimates based on a wider range of parameters in Table 3, showing that we have sufficient power to detect between-group differences in the primary outcome with even smaller effect sizes. Power was calculated using PASS software V15.0 (NCSS), with all analyses conducted with a 2-tailed α of .025, resulting in a family-wise error rate of at most 0.05 for the primary outcome.

For aim 2, we anticipate that there will be no higher than 7% prevalence of undiagnosed HIV. Combined with expected HIV testing rates by 12 months of at least 75%, 45%, and 35% [46,47] in the 3 arms, we anticipate the available sample size of participants without HIV for aim 2 to be CHALO! 2.0 (n_1_=247), AMC (n_2_=139), and DCO (n_2_=108). Based on HST’s current linkage-to-care rates for HIV-negative individuals (30%) [48], we expect the rate of linkage-to-prevention care at 12 months to be 30% for CHALO! versus 15% for AMC vs 5% for DCO. The study’s 2 main effects (CHALO! vs AMC and CHALO! 2.0 vs DCO) have >90% and >99% power, respectively (Table 3). We also provide power estimates for smaller effect sizes. The power was calculated using PASS software V15.0., with a 2-tailed α of .025, resulting in a family-wise error rate of at most 0.05 for the aim 2 primary outcome.

**Table 2:** HIV testing proportion at 6 months.

Power^a^	Intervention (CHALO! 2.0; n_1_=300)	Attention–Matched Control (n_2_=300)	Digital coupon–only (n_2_=300)
>99%	0.5-0.75	0.2-0.5	0.15-0.45
>93%	0.5-0.6	0.35-0.45	0.35-0.45
>59%	0.5-0.60	0.4-0.5	0.4
>15%	0.5	0.45	0.45

^a^Power is for intervention compared with each control separately.

**Table 3:** Proportion linked to HIV prevention by 12 months.

Power^a^	Intervention arm (n_1_=247)	Attention-matched control (n_2_=139)	Digital coupon–only (n_2_=108)
>99%	0.15-0.4	0.05-0.1	0.03-0.1
>98%	0.3-0.4	0.1-0.2	0.3-0.1
>90%	0.3	0.15	0.1-0.15
>70%	0.2	0.1	0.08
>49%	0.3	0.2	0.2
>21%	0.15	0.1	0.1

^a^Power is for intervention compared to each control separately.

### Ethical Considerations

This study has been approved by the IRBs of HST (India) (HST-IRB-42/4-11/2023) and Albert Einstein College of Medicine (United States) (2018-9471) and cleared by the Health Ministry Screening Committee of India.

#### Incentives

Participants receive digital cash incentives for completing research surveys as follows: baseline, INR 300 (US $3.60); 3 months, INR 500 (US $6.01); 6 months, INR 550 (US $6.61); 12 months, INR 600 (US $7.21); and 18 months, INR 1000 (US $12.01). Participants can choose their preferred mode for digital payments (eg, PayTM, GPay, and UPI) which are widely used by all socioeconomic classes in India.

#### Data Management and Security

Data analyses and reporting will be conducted only using deidentified data so that no participant is identifiable, and data will be reported in aggregate. To deliver incentives, participants are asked to provide the mobile numbers linked to the digital payment app (requirement for enrollment and to pay incentives). However, no names or other identifiable information, such as address or locality, are collected. All participant responses are kept confidential. Data collected for the project are downloaded onto secure servers and stored in password-protected, encrypted files.

The study uses a secure survey platform for data collection, using Transport Layer Security encryption to safeguard transmitted data. The survey platform ensures data integrity and confidentiality by adhering to rigorous security measures including FISMA (Federal Information Security Management Act) [49], Federal Information Processing Standards (FIPS) Publication 200 [50], and HITECH (Health Information Technology for Economic and Clinical Health Act) guidelines [51]. Access to the complete dataset is limited to the principal investigator (VVP) and essential study personnel. User-specific access levels and monitoring capabilities are in place to identify any unusual activities within the system. WhatsApp, with its end-to-end encryption, ensures secure communication between the study team and participants. Our technology consultants provide ongoing advice on data security. Study participants are also informed about recorded communications during the consent process.

#### Adverse Events

Possible adverse events include potential breaches of confidentiality leading to embarrassment or disclosure of sexual orientation or participation in an HIV prevention program. Other possible adverse events could include distress if receiving a positive sexually transmitted infection or HIV test result, and negative interactions with nonstudy personnel at care sites not under the purview of HST or the study (eg, private laboratories or non-HST clinics). To mitigate these risks, we continually monitor data security and work only with deidentified data (except when needing to contact participants). Adverse events are monitored during study assessments and participant interactions, with immediate intervention following HST protocols and reporting to the IRB as necessary.

#### Data Safety and Monitoring Board

The study also has a Data Safety and Monitoring Board, which performs biannual safety checks and yearly efficacy analyses. If prespecified significant differences in HIV testing or care linkage are observed, the study arm that does not show benefit may be stopped with the individuals being rerandomized to the other 2 arms.

## Results

The study was funded in 2020, with recruitment starting in April 2022 due to delays from the COVID-19 pandemic. Baseline survey data collection began in April 2022, with follow-up surveys starting in July 2022. As of April 2022, we enrolled 1004 participants in the study. The completion of follow-up data collection is expected in January 2025, with results to be published thereafter.

## Discussion

### Principal Findings

This study aims to assess the effectiveness of a social media–based HIV prevention intervention to increase HIV testing among MSM reached through internet-based platforms in Mumbai, India. This trial uses multiple social media platforms to reach, engage, and deliver digital content to influence HIV testing and linkage-to-care among MSM in India. Globally and in India, unknown HIV status remains a critical barrier to ending the HIV epidemic. Existing interventions to increase HIV testing and facilitate linkage-to-care in India have had limited success in reaching MSM who do not visit MSM-associated physical venues. In addition, existing interventions that rely on physical venue-based outreach may not adapt to the shifting patterns of socialization and partner seeking from offline to digital venues. Consequently, alternative outreach and engagement models, such as CHALO! 2.0, are necessary to engage MSM unreached by current HIV testing and care programs.

If successful, CHALO! 2.0 has the potential to overcome several multilevel barriers to HIV testing and status-neutral linkage to care for Indian MSM. It may efficiently reach MSM online, overcome barriers to engagement, and facilitate linkage to multiple HIV testing and care options that are consistent with individual preferences and meet individual needs [52-55]. We anticipate findings from this study will advance our understanding of how an SMT-mediated multilevel intervention can influence care outcomes for stigmatized conditions such as HIV in low- and middle-income country settings. We also expect that the study design will help determine the extent to which an HIV-focused educational intervention may be needed to increase HIV testing and care uptake relative to control conditions.

### Strengths and Limitations

The CHALO! 2.0 intervention has several key strengths. First, it leverages WhatsApp, a widely-used social media and messaging platform to engage Indian MSM and overcome challenges to adopting new technology or apps—a barrier faced by many eHealth interventions. Second, CHALO! 2.0 intervention components are theoretically grounded in multiple behavior change frameworks, thereby increasing its likelihood of success for increasing HIV testing and linkage-to-care. Next, CHALO! 2.0 offers tailored and community-developed digital content addressing the unique needs of Indian MSM, fostering a more comprehensive approach. Finally, providing access to a range of free HIV testing and care options including self-testing and bypassing in-person counseling for testing may help promote privacy and comfort and overcome anticipated stigma—an important barrier to HIV testing engagement.

This study also has limitations to consider. First, the study findings may not generalize to Indian MSM not reachable online, for whom face-to-face interventions currently exist. Next, the study’s dependence on digital technology may pose challenges for individuals with low digital literacy, potentially hindering their engagement or comprehension of the information provided. Finally, the study design relies on the ability to read and write to successfully enroll into the study; therefore, different strategies and study designs are needed to engage individuals with low literacy.

### Conclusion

Global health agencies have called for digital health interventions to engage populations who are vulnerable to HIV but are not being reached, yet few impactful and scalable models exist for low- and middle-income countries. In India, with one of the world’s largest MSM HIV epidemics, no such eHealth interventions have been rigorously designed—a gap this study will help fill. This study has the potential to provide actionable information to diverse stakeholders, including whether or not CHALO! 2.0 is effective relative to the control conditions and insights about virtually reaching and engaging Indian MSM. This study addresses key evidence gaps by testing an innovative, scalable, mHealth-based intervention model to engage MSM in India at a high priority for HIV-related interventions. Finally, it has the potential to provide policy-relevant information for government and service organizations to design and implement similar mHealth-based interventions to address HIV.

## Appendix

Multimedia Appendix 1 Peer-review report for the Chalo! 2.0 protocol.

## Abbreviations

AMC: attention-matched control
CAB: community advisory board
DCO: digital coupon–only
FIPS: Federal Information Processing Standards
FISMA: Federal Information Security Management Act
HCW: health care worker
HITECH: Health Information Technology for Economic and Clinical Health Act
HST: The Humsafar Trust
IMB: information-motivation-behavioral skills
IRB: institutional review board
LGBTQ: lesbian, gay, bisexual, transgender, and queer or questioning
LGBTQ+: lesbian, gay, bisexual, transgender, queer or questioning, plus (others)
mHealth: mobile health
MSM: men who have sex with men
NACO: National AIDS Control Organization
PEP: postexposure prophylaxis
PrEP: pre-exposure prophylaxis
SEM: socio-ecological model
SMT: social media technology
UNAIDS: Joint United Nations Programme on HIV/AIDS
UPI: Unified Payments Interface

## Data Availability

The datasets generated and analyzed during the current study will be made available after study completion and publication of primary findings from the corresponding author on reasonable request with appropriate ethical approvals and data use agreements.
