# Supplementary material for: The CHALO! 2.0 mHealth-Based Multilevel Intervention to Promote HIV Testing and Linkage-to-Care Among Men Who Have Sex with Men in Mumbai, India: Protocol for a Randomized Controlled Trial
Source: JMIR Res Protoc. 2024 Nov 5;13:e59873. doi: 10.2196/59873 (PMC11576599; doi:10.2196/59873)
Supplement: Multimedia Appendix 1 [file resprot_v13i1e59873_app1.pdf]

**SUMMARY STATEMENT**

| | | | |
|---|---|---|---|
| **PROGRAM CONTACT:** | **( Privileged Communication )** | ***Release Date:*** | **07/25/2019** |
| | | ***Revised Date:*** | |

***Application Number:*** **1 R01 MH119001-01A1**

**Principal Investigator**

**PATEL, VIRAJ V**

**Applicant Organization: ALBERT EINSTEIN COLLEGE OF MEDICINE**

***Review Group:*** **PPAH**
**Population and Public Health Approaches to HIV/AIDS Study Section**
**AIDS - EXP. REV.**

| | | | |
|---|---|---|---|
| ***Meeting Date:*** | **07/15/2019** | ***RFA/PA:*** | **PA18-278** |
| ***Council:*** | **OCT 2019** | ***PCC:*** | **9A-ASGT** |
| ***Requested Start:*** | **09/01/2019** | | |
| | | ***Dual IC(s):*** | **NR** |

| | |
|---|---|
| ***Project Title:*** | **CHALO!: A mobile technology based intervention to accelerate HIV testing and linkage to prevention and treatment** |
| ***SRG Action:*** | **Impact Score:34 Percentile:16** |
| ***Next Steps:*** | **Visit https://grants.nih.gov/grants/next_steps.htm** |
| **Human Subjects:** | **30-Human subjects involved - Certified, no SRG concerns** |
| **Animal Subjects:** | **10-No live vertebrate animals involved for competing appl.** |
| **Gender:** | **3A-Only men, scientifically acceptable** |
| **Minority:** | **5A-Only foreign subjects, scientifically acceptable** |
| **Age:** | **3A-No children included, scientifically acceptable** |

**1R01MH119001-01A1 PATEL, VIRAJ**

**EARLY STAGE INVESTIGATOR**

**NEW INVESTIGATOR**

**RESUME AND SUMMARY OF DISCUSSION:** The applicants seek to test the efficacy of a social media-based Technology (SMT) intervention in combination with coupon for free HIV testing to promote HIV testing and PrEP for MSM who test HIV negative and engagement in care for MSM who test positive in Mumbai, India. This is a very important population to focus on as their rate of HIV infection continues to be among the highest in the world. The use of social media as a vehicle for the intervention is appropriate as these are means with which this population is familiar. CHALO!2.0 as this intervention is labeled will be compared to two control conditions: an attention-matched SMT-based control (also including a digital coupon for free HIV testing), and a digital coupon only control to determine if it will increase HIV testing and linkage-to-care in sexually active MSM who are unaware of their HIV status and are recruited online. This intervention is innovative being among the first to use an online intervention among MSM in India. This resubmission was very responsive to prior critiques; the applicants have, among other things, clarified how they will produce messaging content for the intervention; they have expanded the theoretical grounding of the intervention; have refined their inclusion criteria to MSM who have engaged in anal sex with other men in the past year, and have strengthened the reliability of the measures they capture by instituting some checks against fraudulent participation. The application is highly improved; there were some remaining weaknesses that slightly reduced enthusiasm for the application. While the applicants have provided strong justification for not implementing home-based self-testing, referring participants to a clinic for testing or self-testing poses its own set of problems (e.g., transportation) that may limit access to care. Despite, these weaknesses, the committee thought this project very scalable and a strong intervention with the potential for having high impact on the sexual health of MSM in India.

**DESCRIPTION (provided by applicant):** Despite effective HIV prevention and treatment, the global HIV epidemic continues to grow. Current programs to engage populations at high HIV risk have had limited impact, in part because they do not reach those at highest risk. To reduce the global burden of HIV, new, evidence-based, far-reaching strategies are needed to engage individuals at risk in HIV prevention and treatment. In the U.S. and globally, people are increasingly using mobile/social technologies (SMT) (e.g. dating apps, WhatsApp, Facebook) to socialize and meet partners. While this shift in how individuals socialize has disrupted traditional face-to-face outreach, SMT provides an unprecedented opportunity to engage historically hard-to-reach groups, such as men who have sex with men (MSM), for HIV prevention and treatment. Th goal of this study is to test whether a SMT-based behavioral intervention that includes a digital coupon for free HIV testing (CHALO! 2.0) enhances HIV testing and linkage-to-care among a population that continues to experience high rates of HIV infection in India. The scientific premise of this proposal is that a community-developed, theory-based intervention will engage MSM at high risk of HIV who are reached online, and that the intervention will increase HIV knowledge and enhance motivation and behavioral skills to obtain HIV testing and get linked to HIV prevention (if HIV- uninfected) or HIV treatment (if HIV-infected). The rationale for this research study is that determining efficacy of a rapidly scalable SMT-based behavioral intervention is needed before widespread dissemination. The significance of this research question is that even a modestly effective SMT-based HIV prevention intervention may efficiently reach many individuals at

high risk of HIV and lead to increased HIV status awareness, better linkage-to-care, and fewer new HIV infections. Methods: In a pragmatic randomized controlled trial, we will determine whether CHALO! 2.0 results in increased HIV testing and linkage-to-care (prevention or treatment) among sexually active MSM who are unaware of their HIV status (never tested or tested >6 months ago) and are recruited online. We will conduct a 12-week, three arm randomized trial comparing CHALO! 2.0 to two control conditions: an attention-matched SMT-based control (also including a digital coupon for free HIV testing), and a digital coupon only control. Potential Impact: While global health agencies have called for online interventions to engage populations at high HIV risk who are not being reached, few effective models exist and none exist in India, which is home to one of the world's largest MSM HIV epidemics. This study will address this gap by testing a scalable and replicable online model to engage individuals at high HIV risk and link them to HIV testing and prevention or treatment. This proposal is responsive to NIH PA-18-278: Innovations in HIV Testing…to Optimize HIV Care Continuum Outcomes, and addresses multiple high priority research areas of NIH's Office of AIDS Research. The proposed model, if found effective, may also accelerate achieving UNAIDS 90-90-90 care targets.

**PUBLIC HEALTH RELEVANCE:** Many people at high risk of HIV in the United States and globally are not reached by existing prevention programs. Online interventions can reach and engage individuals into HIV treatment and prevention, but few effective models exist anywhere. Therefore, it is important to develop clinically proven online prevention programs to engage people at high HIV risk and prevent new infections.

**CRITIQUE 1**

Significance: 3
Investigator(s): 2
Innovation: 3
Approach: 3
Environment: 1

**Overall Impact:** This R01 application is a resubmission of a proposal for a 3-group pragmatic randomized controlled trial to assess the effects of a social media mobile technology intervention (CHALO! 2.0) to increase HIV testing and linkage to care among MSM in Mumbai, India. The promising results of the open-label pilot study testing the intervention provide a strong scientific premise for this trial, which will enroll 1,000 MSM who are unaware of their HIV status. Participants will complete an online survey and be randomized to one of three arms: (1) attention-matched control that includes a digital coupon for HIV testing; (2) CHALO! 2.0, which also includes a digital coupon for HIV testing; or (3) only a digital coupon for free HIV testing. The digital coupon will allow participants to select one of three HIV testing approached: (1) standard HIV counseling and testing at a site operated by Humasafar Trust (HST)—a partner organization that provides services to LGBTQ across India; (2) self-testing with standard pre- and post-test counseling at an HST site; or (3) HIV testing at a private lab. The CHALO! 2.0 intervention will include 12 weeks of twice weekly digital communications through WhatsApp that are tailored to social ecological model (SEM) and Information Motivation and Behavior Skills (IMB) model domains. Participants will be followed for 18 months after enrollment to assess intervention outcomes. This is a strong application by an excellent team of investigators. MSM suffer disproportionately from HIV in India, and HIV testing rates among MSM are relatively low, therefore the proposed intervention to increase HIV testing among MSM in India addresses a significant problem. The Approach includes many strengths. However, the descriptions of the HIV self-testing at HST sites and the standard HIV testing at HST sites seem very similar because both groups will receive standard HIV pre- and post-test counseling. Additionally, although the application uses a "status neutral" approach that addresses the needs of MSM who test negative for HIV as well as those who test

positive, the description of activities for linking participants who test negative to PrEP is relatively thin compared with the description of linkage to care for participants who test positive. In this resubmission, the applicants satisfactorily addressed most of the issues raised in the original review.

**1. Significance:**

**Strengths**

- HIV testing rates in India are relatively low among MSM who are disproportionately infected with HIV, and the proposed intervention has the potential to increase HIV testing among them.
- Because of stigma, many MSM in India do not seek HIV testing. The social media mobile technology (SMT) intervention that will be tested in this study has the potential to reach MSM who may be difficult to reach through in-person methods and link them to an HIV testing method of their choice.
- The SMT intervention has the potential to be scaled-up if it demonstrates efficacy in this study.
- The scientific premise that the SMT intervention will increase HIV testing and linkage to care among MSM in a setting like India seems justified.

**Weaknesses**

- The applicants make a strong case for why home-based HIV self-testing cannot be used. Nonetheless, forcing participants to go to a physical site for testing may limit the reach of the CHALO! 2.0 intervention that is otherwise delivered completely online.

**2. Investigator(s):**

**Strengths**

- The investigators have considerable experience conducting similar studies with MSM in Mumbai.
- The team includes expertise in developing and testing interventions using social media and mobile technologies.
- The team has expertise in conducting pragmatic randomized trials.

**Weaknesses**

- None noted.

**3. Innovation:**

**Strengths**

- Allowing participants to select from 3 HIV testing strategies is innovative.
- The status neutral intervention approach that links participants to appropriate care based on their HIV status is relatively innovative.
- The social media mobile technology intervention that includes structural components in the form of digital coupons for free HIV testing is innovative.

**Weaknesses**

- These innovations are modest.

**4. Approach:**

**Strengths**

- The preliminary study that demonstrated the feasibility and acceptability of the proposed intervention is a strength.
- The 3-group pragmatic randomized controlled trial design with urn randomization provides a high degree of scientific rigor for study.
- The digital coupons for HIV testing increase the rigor of the study by making it easier to track where and when participants choose to get tested for HIV.
- The community advisory board is a strength.
- Using the Information-Motivation-Behavior and Skills (IMB) framework and the social ecological model to inform the mHealth component of the intervention is a strength.
- The plans for measuring and preventing fraudulent participation in the study are a strength.
- The multiple follow-up interviews at the 3, 6, 13, and 18 months post-enrollment are a strength.
- The application focuses on MSM, but it does not specifically address sex as a biological variable. The eligibility criteria include "identify as male" and "anal sex with men in the past year." These eligibility criteria do not necessarily exclude biological women/females.

**Weaknesses**

- The difference between the HIV self-test and the standard HIV test at HST seems minimal because it sounds like participants in both testing formats will receive standard pre- and post-test HIV counseling from a counselor. It is possible that the pre- and post-test counseling for the self-testers will be included in the instructional video online and post-test self-administered survey, but that is not clear.
- The application uses a "status neutral" approach, but the component for linking HIV negative participants to PrEP is not as developed as the component for linking HIV positive participants to HIV treatment. The applicants are experienced in linking HIV negative MSM to PrEP and this outcome will be measured, but the application includes relatively few details regarding linkage to PrEP.

**5. Environment:**

**Strengths**

- The partnerships with Humsafar Trust and with the private laboratory (Suburban Diagnostics) are a strength.

**Weaknesses**

- None noted.

**Study Timeline**

**Strengths**

- The timeline includes 9 months for startup activities (i.e. obtaining IRB approval, hiring and training staff, finalizing protocols and measures and registering with Clinicaltrials.gov. The allocated time should be sufficient to complete these activities.

- The timeline includes approximately 15 months for recruitment with an anticipated flow of 70 to 100 participants per month. It also includes time for follow-up visits at 6, 12, and 18 months.

- The timeline includes other important project activities, and the times periods allocated for each activity are reasonable.

**Weaknesses**

- For the 18-month follow-ups, the timeline does not seem to include any additional time beyond 18 months. However, some additional time may be needed to complete follow-up interviews with people who are difficult to find and interview on their scheduled date. However, this is a minor weakness.

**Protections for Human Subjects**

Acceptable Risks and/or Adequate Protections

- The use of Qualtrics, REDCap and WhatsApp for online data collection and intervention provides adequate safeguards and encryption for protecting online information.

**Data and Safety Monitoring Plan (Applicable for Clinical Trials Only):**

Acceptable

- The DSMP includes a DSMB and appropriate safeguards.

**Inclusion Plans**

- Sex/Gender: Distribution justified scientifically.
- Race/Ethnicity: Distribution justified scientifically.
- For NIH-Defined Phase III trials, Plans for valid design and analysis: Scientifically acceptable.
- Inclusion/Exclusion Based on Age: Distribution justified scientifically.
- The project works only with MSM so it is justified in excluding women. The expected race/ethnicity of the sample is justified because it is similar to the racial/ethnic distribution of MSM in Mumbai. The study is justified in excluding children under 18 because there are different policies and regulations in India that apply to HIV testing and for children under 18.

**Vertebrate Animals**

Not Applicable (No Vertebrate Animals)

**Biohazards**

Not Applicable (No Biohazards)

**Resubmission**

- The applicants addressed most of the issues raised in critiques by reviewers of the previous submission.

**Applications from Foreign Organizations**

Justified

- The study will be conducted Mumbai, India.

**Select Agents**

Not Applicable (No Select Agents)

**Resource Sharing Plans**

Acceptable

- The applicants will create a deidentified dataset, and they will make it available for secondary to other researchers who submit a data sharing request.

**Authentication of Key Biological and/or Chemical Resources**

Not Applicable (No Relevant Resources)

**Budget and Period of Support**

Recommend as Requested

**CRITIQUE 2**

Significance: 2
Investigator(s): 3
Innovation: 1
Approach: 3
Environment: 1

**Overall Impact:** This is a proposal for three-arm pragmatic RCT to increase linkage to HIV testing among MSM in India. The proposal is significant in that the population is large and disproportionately impacted by HIV in India. The project is well informed by previous pilot work in CHALO 1.0. The intervention is testing two different approaches using social media and mobile phones to reach and increase HIV testing among MSM – with all three with potentially high relevance and scalability in India. The partnerships developed with Humsafar trust are longstanding and built on many years of collaboration- Humsafar trust is a strong collaborator with a track record of advocacy and research with MSM in Mumbai. There does seem to be a missed opportunity for more engagement with PrEP uptake and persistence. The investigators have thoughtfully crafted the intervention approach to address stigma, which includes some limitations to design like having home testing at the Humsafar Trust site; however, this somewhat defeats the purpose of offering self-testing. There also appears to be trans men included but there is no particular focus on tailoring to their unique needs. Also, trans men will be included in the project, but there was no articulation of their unique needs for recruitment online or the barriers they may face for HIV testing or using services at an organization geared towards MSM in India. Despite minor weaknesses, this proposal has significant chance for impact on the HIV epidemic and molding new ways to effectively reach MSM and increase their rates of HIV testing.

**1. Significance:**

**Strengths**

- If this intervention increases engagement in HIV testing and regular testing, it could have a large impact on the Indian HIV epidemic that is high among MSM.

- Same sex behavior was until very recently criminalized in India, making many MSM hidden. This intervention has the chance of reaching the many (millions) of MSM who are hidden or simply do not attend venues or visit NGOs for gay men.
- This intervention uses technologies native to MSM in India and can be translated for use on other platforms, providing promise for scalability if found to be efficacious.
- Investigators are addressing structural stigma by offering options for HIV testing outside the MSM partner NGO including private labs and self-testing.

**Weaknesses**

- CHALO 1.0 was completed four years ago and it is not clear whether the preliminary efficacy of the intervention resulted in the intervention implementation or scaling at HST or elsewhere in India. Now investigators propose to test the 2.0 version, which will take another 5 years until results are released. Given the immediacy of the needs for HIV prevention among MSM in India, the long timeframe and lack of clarity on community benefit so far are a challenge to the significance of the proposal.

**2. Investigator(s):**

**Strengths**

- Dr. Patel is an excellent PI for this proposal; he brings community-based experience and a strong history of investigator collaboration and working with the local NGO in India, Humsafar trust.
- Overall Dr. Patel has developed a strong team with mostly necessary investigators for the study design, implementation and analysis phases of the research.

**Weaknesses**

- It is not entirely clear why three of Dr. Patel's K23 mentors are listed as investigators as much of the support they would lend is similar (e.g., protocol and measures development, dissemination). It seems like the time is ripe for this early stage investigator to branch out independent from his many mentors.

**3. Innovation:**

**Strengths**

- Approach to move beyond in person outreach to increase linkage to HIV testing and care is novel and moves the field forward in India.
- Three arm trial design offers the chance for a very simple intervention (digital coupon) to be tested in addition to the more comprehensive CHALO 2.0 intervention, which offers novel data on more than one technology-based intervention to be proven efficacious.

**Weaknesses**

- None noted.

**4. Approach:**

**Strengths**

- Strong track record of recruitment of large numbers of MSM and of retention.
- Intervention design addresses stigma, which is a critical factor to engaging MSM in India into HIV prevention and care.

- Strong rationale for why investigator does not anticipate fraudulent online participation and how the team will monitor and validate participants.
- Three-arm pragmatic RCT design offers advantages over a traditional intervention and control arm study where failure of the main intervention leaves the study with little to no contributions to the science. These two interventions may both have promise and benefit for the MSM community in Mumbai, increasing the utility of the overall project results.

**Weaknesses**

- How is the HIV self-testing and private lab testing provided for free? Are all clinics in Mumbai participating? Will this be sustainable after the study?
- Inclusion of Facebook and Insta as recruitment sites may be problematic as they will be much more likely to reach general audiences where risk to fraudulent participation increases.
- HIV testing sites and stigma reduction is very limited by the fact that 2 of the three HIV testing sites are at HST. It is unclear how many private lab testing sites are available, and if the number of private sites is small, stigma may still be an issue as these sites will become associated with HIV testing for MSM in Mumbai.

**5. Environment:**

**Strengths**

- Humsafar trust is a strong Indian partner with a substantial history of work and research with MSM in India.

**Weaknesses**

- None noted.

**Study Timeline**

**Strengths**

- Given the short timeframe for intervention, the timeline for the activities, recruitment and follow-up seems feasible, especially given this intervention is building off prior work, including existing protocols.

**Weaknesses**

- Timeline for IRB approval seems ambitious given there are two IRBs. Will likely need to get approval.
- Research to practice time period is short.

**Protections for Human Subjects**

Acceptable Risks and/or Adequate Protections

**Data and Safety Monitoring Plan (Applicable for Clinical Trials Only):**
Acceptable

**Inclusion Plans**

- Sex/Gender: Distribution justified scientifically.
- Race/Ethnicity: Distribution justified scientifically.
- For NIH-Defined Phase III trials, Plans for valid design and analysis: Scientifically acceptable.
- Inclusion/Exclusion Based on Age: Distribution justified scientifically.

**Vertebrate Animals**
Not Applicable (No Vertebrate Animals)

**Biohazards**
Not Applicable (No Biohazards)

**Resubmission**

- The team was responsive to many of the critiques of the prior reviewers, especially the response to the need for more details on the social media components for the CHALO 2.0 arm, which are much more specific than in the last proposal. However, the control components still lack specificity.

**Applications from Foreign Organizations**
Not Applicable (No Foreign Organizations)

**Select Agents**
Not Applicable (No Select Agents)

**Resource Sharing Plans**
Acceptable

**Authentication of Key Biological and/or Chemical Resources**
Not Applicable (No Relevant Resources)

**Budget and Period of Support**
Recommend as Requested

**CRITIQUE 3**

Significance: 1
Investigator(s): 1
Innovation: 3
Approach: 4
Environment: 1

**Overall Impact:** This resubmitted R01 by a new investigator proposes the development, and examination of the feasibility and acceptability of HIV testing through an SMT intervention (CHALO!2.0) among Indian MSM (N=1000). The scientific premise and study's significance are well justified given high rates of HIV among this population, with 7% prevalence among MSM in Mumbai, the study context. The investigative team is strong with a junior PI being supported by exceptional senior colleagues. The PI has conducted preliminary studies and collaborated with the study team's previous tailored HIV testing support. The preliminary data directly supports the proposed study. The proposal was extremely responsive to the prior reviews, but several issues persist which are detailed below. Briefly, remaining limitations include: the passive interaction with a virtual peer, the lack of full integration of IMB into the measures, and the study's procedures for linkage to care and the documentation thereof. Although minor, it is curious that there are only 2 study aims, given the scope of an R01 and the proposed study. The study still has a number of strengths and could be an extensive contribution, with a potential for high impact.

**1. Significance:**

**Strengths**

- The study population is significant, with high HIV rates among MSM in India as well as a lack of awareness of HIV status.
- The use of an SMS intervention with a virtual peer could be a significant contribution to the literature and practice, in many settings.
- The study is grounded in the investigators' prior relevant research.

**Weaknesses**

- None noted.

**2. Investigator(s):**

**Strengths**

- The PI is new and has a promising career, he is supported by an excellent team of seasoned researchers.

**Weaknesses**

- None noted.

**3. Innovation:**

**Strengths**

- The range of 3 options for HIV testing and providing somewhat of a natural experiment to examine preferences and correlates thereof.

**Weaknesses**

- Although the intervention components are not particularly innovative in and of themselves, they are in the context of this intervention targeted MSM in India.

**4. Approach:**

**Strengths**

- Collaboration with a well-established, community-based MSM focused health organization will potentiate the success of the study and future impact in being implemented. The provision of the intervention in part in the HST clinic is a strength.
- The use of SMT is a strong intervention approach, informed by a theoretical framework and pilot data and formative work.
- The inclusion of and meaningful description of the CAB will help the relevance of the study design.
- The inclusion of men only is appropriate.
- The coupon system is an efficient way to collect relevant HIV testing and treatment information.
- The methods are somewhat rigorous.

**Weaknesses**

- Given the context of an RCT and the focus on a "virtual" peer, it is curious that there is only the potential for peer contact (e.g., the participant has to reach out) rather than having contact being a proactive component – at least once – so that it could be measured..
- If participants test positive for HIV, how will their messages change to support access to care and adherence?
- Who will record the brief videos and how will their topics be selected vs. text messages?
- Aim 2 reads as if linkage to care, ART uptake is a part of the main outcome and not the behavioral outcomes listed in the Aim 2 analysis. Given the distinction between relevant outcomes for HIV+ and HIV-participants, this could have been better articulated. Given the N and the potential for 70 HIV+ individuals, why isn't this a main outcome for this subset?

**5. Environment:**

**Strengths**

- The domestic and foreign research environments are strong and can support the proposed work.

**Weaknesses**

- None noted.

**Study Timeline**

**Strengths**

- The timeline is sufficient with ample time to conduct formative work, develop the intervention, and complete the evaluation.

**Weaknesses**

- None noted.

**Protections for Human Subjects**

Acceptable Risks and/or Adequate Protections

**Data and Safety Monitoring Plan (Applicable for Clinical Trials Only):**

Acceptable

**Inclusion Plans**

- Sex/Gender: Distribution justified scientifically.
- Race/Ethnicity: Distribution justified scientifically.
- For NIH-Defined Phase III trials, Plans for valid design and analysis:
- Inclusion/Exclusion Based on Age: Children are appropriately excluded; only adults of all ages are included

**Vertebrate Animals**

Not Applicable (No Vertebrate Animals)

**Biohazards**

Not Applicable (No Biohazards)

**Resubmission**

- The resubmission was responsive; adding detail about how the intervention will be developed, providing additional information about the study's innovation, tightening the inclusion criteria, clarifying the linkage to care plans, and expanding the theoretical model

**Applications from Foreign Organizations**

Justified

- The study is significant and would be not possible without local partners conducting on-site.

**Select Agents**

Not Applicable (No Select Agents)

**Resource Sharing Plans**

Not Applicable (No Relevant Resources)

**Authentication of Key Biological and/or Chemical Resources**

Not Applicable (No Relevant Resources)

**Budget and Period of Support**

Recommend as Requested

**THE FOLLOWING SECTIONS WERE PREPARED BY THE SCIENTIFIC REVIEW OFFICER TO SUMMARIZE THE OUTCOME OF DISCUSSIONS OF THE REVIEW COMMITTEE, OR REVIEWERS' WRITTEN CRITIQUES, ON THE FOLLOWING ISSUES:**

**PROTECTION OF HUMAN SUBJECTS: ACCEPTABLE**

**INCLUSION OF WOMEN PLAN (3A): ACCEPTABLE**

**INCLUSION OF MINORITIES PLAN (M5A): ACCEPTABLE**

**INCLUSION ACROSS THE LIFESPAN PLAN (7A): ACCEPTABLE**

**COMMITTEE BUDGET RECOMMENDATIONS: The budget was recommended as requested.**

---

Footnotes for 1 R01 MH119001-01A1; PI Name: Patel, Viraj V

NIH has modified its policy regarding the receipt of resubmissions (amended applications). See Guide Notice NOT-OD-14-074 at http://grants.nih.gov/grants/guide/notice-files/NOT-OD-14-074.html. The impact/priority score is calculated after discussion of an application by averaging the overall scores (1-9) given by all voting reviewers on the committee and multiplying by 10. The criterion scores are submitted prior to the meeting by the individual reviewers assigned to an application, and are not discussed specifically at the review meeting or calculated into the overall impact score. Some applications also receive a percentile ranking. For details on the review process, see http://grants.nih.gov/grants/peer_review_process.htm#scoring.

**MEETING ROSTER**

**Population and Public Health Approaches to HIV/AIDS Study Section**
**AIDS and Related Research Integrated Review Group**
**CENTER FOR SCIENTIFIC REVIEW**
**PPAH**
**07/15/2019 - 07/16/2019**

**Notice of NIH Policy to All Applicants:** Meeting rosters are provided for information purposes only. Applicant investigators and institutional officials must not communicate directly with study section members about an application before or after the review. Failure to observe this policy will create a serious breach of integrity in the peer review process, and may lead to actions outlined in NOT-OD-14-073 at https://grants.nih.gov/grants/guide/notice-files/NOT-OD-14-073.html and NOT-OD-15-106 at https://grants.nih.gov/grants/guide/notice-files/NOT-OD-15-106.html, including removal of the application from immediate review.


**CHAIRPERSON(S)**

ALLEN, SUSAN A, MD
PROFESSOR
DEPARTMENT OF PATHOLOGY
AND LABORATORY MEDICINE
SCHOOL OF MEDICINE
EMORY UNIVERSITY
ATLANTA, GA 30322

**MEMBERS**

AMIRKHANIAN, YURI A, PHD *
PROFESSOR
DEPARTMENT OF PSYCHIATRY AND BEHAVIORAL MEDICINE
CENTER FOR AIDS INTERVENTION RESEARCH
MEDICAL COLLEGE OF WISCONSIN
MILWAUKEE, WI 53202

BAETEN, JARED, MD, PHD
PROFESSOR AND VICE CHAIR
DEPARTMENT OF GLOBAL HEALTH
PROFESSSOR, DEPARTMENT OF EPIDEMIOLOGY
SCHOOL OF PUBLIC HEALTH
UNIVERSITY OF WASHINGTON
SEATTLE, WA 98104

BAUERMEISTER, JOSE ARTURO, PHD
PROFESSOR
DEPARTMENT OF FAMILY AND COMMUNITY HEALTH
SCHOOL OF NURSING
UNIVERSITY OF PENNSYLVANIA
PHILADELPHIA, PA 19104

BAUMAN, LAURIE J, PHD
PROFESSOR
DEPARTMENT OF PEDIATRICS
ALBERT EINSTEIN COLLEGE OF MEDICINE
BRONX, NY 10461

BOOTH, ROBERT EDWIN, PHD *
PROFESSOR
DEPARTMENT OF PSYCHIATRY
SCHOOL OF MEDICINE
UNIVERSITY OF COLORADO
DENVER, CO 80218

BRAITHWAITE, RONALD SCOTT, MD *
CHIEF AND INTERIM DIRECTOR
DEPARTMENT OF POPULATION HEALTH
SCHOOL OF MEDICINE
NEW YORK UNIVERSITY
NEW YORK, NY 10016

COOK, ROBERT L, MD, MPH
PROFESSOR
DEPARTMENT OF EPIDEMIOLOGY
COLLEGE OF MEDICINE
UNIVERSITY OF FLORIDA
GAINESVILLE, FL 32610

DICKSON-GOMEZ, JULIA B, PHD
PROFESSOR
CENTER FOR AIDS INTERVENTION RESEARCH
DEPARTMENT OF PSYCHIATRY AND BEHAVIORAL MEDICINE
MEDICAL COLLEGE OF WISCONSIN
MILWAUKEE, WI 53202

DOMBROWSKI, KIRK, PHD *
JOHN BRUHN PROFESSOR OF SOCIOLOGY
DEPARTMENT OF SOCIOLOGY
COLLEGE OF ARTS AND SCIENCES
UNIVERSITY OF NEBRASKA-LINCOLN
LINCOLN, NE 68588

FUJIMOTO, KAYO, PHD *
ASSOCIATE PROFESSOR
DEPARTMENT OF HEALTH PROMOTIONS
AND BEHAVIORAL SCIENCES
SCHOOL OF PUBLIC HEALTH
UNIVERSITY OF TEXAS AT HOUSTON
HOUSTON, TX 77030

HABERER, JESSICA ELIZABETH, MD
ASSOCIATE PROFESSOR
DEPARTMENT OF MEDICINE
MASSACHUSETTS GENERAL HOSPITAL
BOSTON, MA 02114

HAHN, JUDITH ALISSA, PHD *
PROFESSOR
DEPARTMENT OF MEDICINE
SAN FRANCISCO GENERAL HOSPITAL
UNIVERSITY OF CALIFORNIA, SAN FRANCISCO
SAN FRANCISCO, CA 94143

HIGHTOW-WEIDMAN, LISA B, MD, MPH
PROFESSOR
INSTITUTE FOR GLOBAL HEALTH
AND INFECTIOUS DISEASES
GILLINGS SCHOOL OF PUBLIC HEALTH
UNIVERSITY OF NORTH CAROLINA
CHAPEL HILL, NC 27599-7030

JENNESS, SAMUEL, PHD *
ASSISTANT PROFESSOR
DEPARTMENT OF EPIDEMIOLOGY
ROLLINS SCHOOL OF PUBLIC HEALTH
EMORY UNIVERSITY
ATLANTA, GA 30030

KELLY, BRIAN C, PHD *
PROFESSOR
DEPARTMENT OF SOCIOLOGY
PURDUE UNIVERSITY
WEST LAFAYETTE, IN 47907

KERSHAW, TRACE S, PHD
PROFESSOR
CENTER FOR INTERDISCIPLINARY RESEARCH ON AIDS
DEPARTMENT OF EPIDEMIOLOGY
SCHOOL OF PUBLIC HEALTH
YALE UNIVERSITY
NEW HAVEN, CT 06510

LELUTIU-WEINBERGER, CORINA, PHD *
ASSISTANT PROFESSOR
RUTGERS BIOMEDICAL AND HEALTH SCIENCES
SCHOOL OF NURSING
RUTGERS UNIVERSITY
NEWARK, NJ 07101

LEVY, JUDITH A., PHD *
ASSOCIATE PROFESSOR
THE HEALTH POLICY AND ADMINISTRATION DIVISION
SCHOOL OF PUBLIC HEALTH
UNIVERSITY OF ILLINOIS AT CHICAGO
CHICAGO, IL 60612

LI, JIANGHONG, MD *
SENIOR RESEARCH SCIENTIST
INSTITUTE FOR COMMUNITY RESEARCH
CENTER FOR INTERDISCIPLINARY RESEARCH ON AIDS
YALE UNIVERSITY
HARTFORD, CT 06106

LOUE, SANA, JD, PHD *
PROFESSOR
CENTER FOR MINORITY HEALTH
DEPARTMENT OF EPIDEMIOLOGY AND BIOSTATISTICS
SCHOOL OF MEDICINE
CASE WESTERN RESERVE UNIVERSITY
CLEVELAND, OH 44106

MCMAHON, JAMES M, PHD *
ASSOCIATE PROFESSOR
DEPARTMENT OF PUBLIC HEALTH SCIENCE
SCHOOL OF NURSING
UNIVERSITY OF ROCHESTER MEDICAL CENTER
ROCHESTER, NY 14642

MEEK, ERIN, DRPH *
SENIOR RESEARCH SCIENTIST
AIDS OFFICE
SAN FRANCISCO DEPARTMENT OF PUBLIC HEALTH
SAN FRANCISCO, CA 94102

NEWCOMB, MICHAEL E., PHD *
ASSISTANT PROFESSOR
DEPARTMENT OF MEDICAL SOCIAL SCIENCES
FEINBERG SCHOOL OF MEDICINE
NORTHWESTERN UNIVERSITY
CHICAGO, IL 60611

PAGE, KIMBERLY, PHD
PROFESSOR AND CHIEF
DIVISION OF EPIDEMIOLOGY, BIOSTATISTICS,
AND PREVENTIVE MEDICINE
DEPARTMENT OF INTERNAL MEDICINE
UNIVERSITY OF NEW MEXICO HEALTH SCIENCES CENTER
ALBUQUERQUE, NM 87131

PAUL, ROBERT H, PHD *
PROFESSOR AND DIRECTOR
DEPARTMENT OF PSYCHOLOGICAL SCIENCES
MISSOURI INSTITUTE OF MENTAL HEALTH
UNIVERSITY OF MISSOURI, ST LOUIS
ST LOUIS, MO 02906

PHILBIN, MORGAN MARI, PHD *
ASSISTANT PROFESSOR
SOCIOMEDICAL SCIENCES
MAILMAN SCHOOL OF PUBLIC HEALTH
COLUMBIA UNIVERSITY
NEW YORK CITY, NY 10032

RICH, JOSIAH D, MD *
PROFESSOR
DEPARTMENT OF MEDICINE AND EPIDEMIOLOGY
BROWN UNIVERSITY MEDICAL SCHOOL
THE MIRIAM HOSPITAL
PROVIDENCE, RI 02906

ROBERTS, MARK STENIUS, MD *
ASSOCIATE PROFESSOR OF MEDICINE
AND INDUSTRIA ENGINEERING
CHIEF, SECTION OF DECISION SCIENCES AND
CLINICAL SYSTEMS MODELING
UNIVERSITY OF PITTSBURGH SCHOOL OF MEDICINE
PITTSBURGH, PA 15213

SCHNEIDER, JOHN, MD, MPH
ASSOCIATE PROFESSOR
DEPARTMENT OF MEDICINE
UNIVERSITY OF CHICAGO
CHICAGO, IL 60637

SHERMAN, SUSAN GAIL, PHD
PROFESSOR
DEPARTMENT OF HEALTH, BEHAVIOR, AND SOCIETY
SCHOOL OF PUBLIC HEALTH
JOHNS HOPKINS UNIVERSITY
BALTIMORE, MD 21205

SWEAT, MICHAEL D, PHD
PROFESSOR
DEPARTMENT OF PSYCHIATRY
AND BEHAVIORAL SCIENCES
MEDICAL UNIVERSITY OF SOUTH CAROLINA
CHARLESTON, SC 29407

WITTE, SUSAN S, PHD *
PROFESSOR
COLUMBIA UNIVERSITY SCHOOL OF SOCIAL WORK
1255 AMSTERDAM AVENUE
NEW YORK, NY 10027

YOUNG, APRIL MARIE, PHD *
ASSOCIATE PROFESSOR
DEPARTMENT OF EPIDEMIOLOGY
COLLEGE OF PUBLIC HEALTH
UNIVERSITY OF KENTUCKY
LEXINGTON, KY 40536

YOUNG, SEAN, PHD *
ASSOCIATE PROFESSOR AND EXECUTIVE DIRECTOR
DEPARTMENTS OF EMERGENCY MEDICINE AND
INFORMATICS
UNIVERSITY OF CALIFORNIA INSTITUTE FOR
PREDICTION TECHNOLOGY
UNIVERSITY OF CALIFORNIA, IRVINE
LOS ANGELES, CA 90024

ZULE, WILLIAM A, DRPH *
SENIOR HEALTH ANALYST
DEPARTMENT OF HEALTH
AND CRIMINAL JUSTICE RESEARCH
RESEARCH TRIANGLE INSTITUTE INTERNATIONAL
RESEARCH TRIANGLE PARK, NC 27709

## SCIENTIFIC REVIEW OFFICER

GUERRIER, JOSE H, PHD
SCIENTIFIC REVIEW OFFICER
CENTER FOR SCIENTIFIC REVIEW
NATIONAL INSTITUTES OF HEALTH
BETHESDA, MD 20892

## EXTRAMURAL SUPPORT ASSISTANT

DILOY, JONATHAN
EXTRAMURAL SUPPORT ASSISTANT
CENTER FOR SCIENTIFIC REVIEW
NATIONAL INSTITUTES OF HEALTH
BETHESDA, MD 20892

* Temporary Member. For grant applications, temporary members may participate in the entire meeting or may review only selected applications as needed.

Consultants are required to absent themselves from the room during the review of any application if their presence would constitute or appear to constitute a conflict of interest.
